# New York-American Veterinary College (Veterinary Department of the New York State University)

**Published:** 1900-07

**Authors:** 


### NEW YORK-AMERICAN VETERINARY COLLEGE (VETERINARY DEPARTMENT OF THE NEW YORK STATE UNIVERSITY).

THE closing exercises of this institution at the Metropolitan Opera House, in New York City, took place on the evening of June 7, 1900. The week of festivities of the University preceded the closing exercises, and as the first graduates of this new college there was accorded a most generous welcome to the ranks and the most cordial interest shown by the University in her first born from this added department to her other successful schools.

The graduates were as follows: Charles Edward Clifford Atkins, Bridgeport, Conn.; Joseph L. Drexler, Thibodaux, La.; Adolph Eickhorn, J. William Fink, and Daniel Joseph Manjan, New York City, N. Y.; James Edward Norton, Glen Cove, L. I.; James Henry Prophett, Suffield, Conn., and William Arthur Young, Utica, N. Y.

The alumni meeting of the College was held in the College building at 141 West Fifty-fourth street, at 3.30 P. M., President Wm. H. Pendry in the chair. The executive committee reported that the usual alumni prize had been provided, the banquet arranged for, the completion of the work incidental to the presentation of the loving cup to Dean Liautard, and a balance of nearly ninety dollars of the fund subscribed. This sum will be used to prepare a suitable tablet or token to be placed in the College building, commemorative of the silver anniversary of the College.

A unanimous spirit prevailed among those present to join with and sustain in every way the work of the old College as a department of the University and to promote its welfare in all directions. The newly elected officers were urged to continue the work of more completely uniting the graduates of the old institution in effective work for the new department.

Dr. Robert Ellis, one of the ever-faithful alumni, was selected as president of the association for the ensuing year, and his identity with the College as an instructor, his keen interest in the University specially equips him to carry into effect the rapidly developing spirit. He will be ably assisted by Drs. W. H. Lowe and H. D. Hanson, who were chosen vice-presidents of the association. In the re-election of Dr. F. R. Hanson as treasurer there was added the duties of secretary also, which will make this dual position one of much greater work and importance during the year.

Dr. Adolph Eickhorn, of the graduating class, will fill the rôle of librarian.

A strong effort and an appreciative desire were shown to retain the efficient services of Dr. C. E. Clayton as secretary, but his declination was positive.

President Ellis will name later the executive committee for the ensuing year.

The banquet of the association at the Hotel Marlborough at 10.30 P. M. following the commencement exercises was a very pleasant and enjoyable affair. Chancellor McCracken and Dr. Munn, of the University council, were the invited guests, and the earnest words of cheer and interest displayed in the remarks of the chancellor and the more than parental care for the future of the veterinary department were listened to with much pleasure by those present. Dr. Munn, of the council, whose long-continued interest in the field of comparative medicine was well exhibited in his remarks, which showed an appreciative watchfulness over the broader development of the veterinary field.

